# Nonlinear analysis of reinforced concrete buildings with different heights and floor systems

**DOI:** 10.1038/s41598-023-41656-7

**Published:** 2023-09-11

**Authors:** Ayman Abd-Elhamed, Sayed Mahmoud, Khalid Saqer Alotaibi

**Affiliations:** 1 Physics and Engineering Mathematics Department, Faculty of Engineering-Mattaria, Helwan University, Cairo, Egypt; 2 Faculty of Engineering, King Salman International University, South Sinai, El-Tur, Egypt; 3 Department of Civil and Construction Engineering, College of Engineering, Imam Abdulrahman Bin Faisal University, Dammam, Saudi Arabia

**Keywords:** Civil engineering, Natural hazards

## Abstract

Most civil structures exhibit nonlinear behavior during moderate to severe earthquakes. Consequently, inelastic analysis is needed for seismic design. Several dynamic and static analysis methods are available for the assessment and design of engineering structures. Two of the available methods in terms of nonlinear dynamic time history analysis and nonlinear static analysis, which is known as pushover analysis, are employed herein to comprehensively study and investigate the seismic performance of multi-story building structures with different floor systems. Moreover, the study is extended to assess the actual values of the response reduction/modification factor (R-factor) for each building model, then evaluate the values with the code-recommended design values. Three-dimensional finite element building models with 5, 10 and 15 stories are developed for the evaluation process. The advanced computer program ETABS is used for developing and analyzing the buildings considering material and geometrical nonlinearity. A suit of seven earthquake records is considered and scaled according to the ASCE-16 seismic design code to excite the building models. The obtained results evidently reveal that the type of floor slab significantly impacts the seismic response of the building. More specifically, the effects of floor slabs on seismic demands are more evident in low- and mid-rise buildings. In addition, the type of slab system and height of the building have more influence on the response modification factors, especially for low-rise building models.

## Introduction

Building structures incorporate reinforced concrete (RC) slabs that are mainly used in floors and roofs to provide flat surfaces of a thickness substantially smaller than their length and width. Slabs are crucial structural elements that alter the seismic response of RC multi-storey buildings exposed to earthquake shakings where they are responsible for transferring the applied loads to the lateral force-resisting elements and subsequently transferring them to the ground^1–4^. The dynamic behavior of RC buildings to lateral loads is significantly impacted by the in-plane stiffness of the utilized slab system and the way in which the lateral loads are transferred^5^. Conventional Flat, hollow block, and solid RC slabs are commonly used as floors in structures worldwide, including in seismic countries. Due to possessing many advantages in terms of architectural demands, use of available spaces, simplicity of formwork and saving time for construction, the flat slab system is commonly used in building construction as a highly acceptable and attractive structural system^6,7^. Another preferred slab system is the hollow block which is suitable for large spans due to its light weight and the use of hidden beams to support the floor system. One of the most well-known floor systems for structures is the solid slab system. Contrary to the hollow block, the solid slab system is carried by drop beams and covers relatively small spans. Investigating the vulnerability of these structural floor systems to ground shaking has become even more important due to their frequent and widespread use. A number of studies have been performed on flat slab and flat plate systems under earthquake loading and have demonstrated promising results regarding their performance^8–11^. The conducted research works and experience have proved that the inherent flexibility of the flat slab and flat plate, as structural floor systems, gives rise to excessive lateral deformations under seismic loads. Consequently, the performance of such systems under earthquake loading is unsatisfactory and vulnerable to more damage^6^. Seismic considerations for the other different types of slabs are seldom seriously addressed. Mahmoud et al.^12^ studied the behavior of high-rise buildings constructed with various floor systems and their nonlinear response to near-fault ground motions containing forward directivity and fling-step. It is widely believed that lateral displacement is the root cause of damage sustained during earthquakes. Seismic damage that occurred during strong earthquake excitation in reinforced concrete structures can be considered evidence of nonlinear behavior. Therefore, seismic displacement prediction is of paramount importance for evaluating the seismic performance of RC buildings. Işık^13^ conducted a study comparing the seismic and structural parameters of earthquakes in Turkey that had a magnitude of six or greater after the year 1900. Measured and updated acceleration values from the most recent two seismic hazard maps were used in the structural analysis of a reinforced concrete building. The study's findings indicate that there are notable disparities in the anticipated displacement values of the target structure due to structural analysis.

It is crucial that displacements are not underestimated during the design procedure. Linear or nonlinear analysis methods can be adopted to determine the seismic displacement demand. Although linear elastic analysis is an acceptable design approach, nonlinear analysis is preferable to capture the yielding behavior of structures during seismic events. It is worth noting that nonlinear analysis is becoming the most popular analysis tool to determine nonlinear seismic demands for both designing new structures or performing seismic assessment for existing structures^14^. The nonlinear analysis involves explicit consideration of the inelastic behavior of the structure. While the detailed NDTHA is believed to be the most rigorous and accurate analysis procedure to compute seismic demands of structures subjected to different types of earthquakes, it’s not a preferred choice for the practice of common design offices due to its complexities. Moreover, it requires a high computational effort, and the execution of this detailed procedure may not be warranted for all buildings^15^. These shortcomings open up the possibility of using novel simpler methods that can serve as an alternative to assess the inelastic seismic responses with satisfactory accuracy and avoid the exact cumbersome detailed NDTHA procedure. Consequently, POA, as a nonlinear static analysis, provides an attractive simplified performance assessment tool^16^. In addition, it involves low computational time and less computations than nonlinear dynamic analysis^17,18^. The basic concept of pushover analysis is to push the structural model by employing a monotonously increased lateral load pattern that represents the induced inertia forces resulting from ground acceleration^19^. The POA produces a characteristic non-linear force–displacement relationship that includes the capacity spectrum, demand spectrum, and performance point. It is widely recognized that POA simultaneously provides data about the strength capacity in the post-elastic range, global displacement and ductility, lateral stiffness, and failure mechanism of the structure. Recently, several studies were conducted using nonlinear static and dynamic analyses in which the adequacy and deficiency of pushover analysis was evaluated against the complexity associated with NDTHA^20–22^.

Inel et al.^20^ performed a comparative study between nonlinear static and dynamic analyses using 3D existing low- and mid-rise building models to clearly identify advantages, disadvantages and applicability limits following the pre-modern and modern Turkish Seismic Codes. The outcomes of the study showed that POA, as a nonlinear static analysis, provides consistent results up to 1 and 0.75% roof drift ratios which almost respectively match 1.5 and 1% inter-story drift ratios for low- and mid-rise buildings. Beyond these limits, the POA may provide misleading demand estimates. Li et al.^23^ investigated the accuracy and applicability of the POA against NDTH using a three-story RC frame structure by analyzing the discrepancies in the calculated structural responses by the POA and THA in terms of the structural top displacement, the inter-story drift ratio and the curvature of column ends. Lagomarsino and Cattari^24^ employed the POA and NDTHA to evaluate the efficiency of existing historical masonry structures modeled through the equivalent frame model. Işık et al.^25^ utilized target displacements as a means of assessing damage estimation and building performance within the field of performance-based earthquake engineering. In their study, regular mid-rise reinforced concrete buildings were used to generate hybrid models to establish target displacements. As a result of conducting structural analysis, a total of three distinct target displacements have been identified as a consequence of selecting the number of stories and seismic risk as variables. In order to accomplish this objective, structural studies were performed by taking into consideration five distinct tale numbers and sixty distinct PGA values. Experimental studies to examine the precision and suitability of the POA through a correlation with the NDTHA have been verified through a complete collapse shaking table test on a ductile RC frame^26^. Moreover, several research works to investigate the performance of asymmetric-plan buildings, precast segmental concrete bridge columns, and steel moment frames employing dynamic and static analysis have also been performed^27–29^. The use of passive control techniques such as tuned mass dampers^30^, tuned liquid dampers^31^, friction dampers^32^, and others for the purpose of seismic response mitigation has been widely accepted by the engineering community for RC buildings that have various heights and different slab systems. The magnitude of the building's structural footprint and the number of floors it has both play a significant role in determining how it will behave when subjected to the effects of an earthquake. Işık et al.^33^ conducted research to determine how the performance of reinforced concrete buildings was affected when the number of stories and the area of the structural footprint were altered. According to the findings of this study, the building's period, displacement, and target displacement all rise with an increase in the number of floors and the building's footprint size, while the building's stiffness decreases.

Although most of the cited herein research works provide information about the use of nonlinear static and dynamic analysis to investigate the seismic response structures, however, most of the used structures to achieve the static and dynamic analysis are limited to moment resisting frames, low-rise buildings, and historical masonry structures. In addition, limited research works have been carried out to investigate the influence of seismic actions on the seismic response of buildings with different heights and floor systems. This paper intends to examine the behaviour of multi-storey RC building structures having different types of floor systems, solid, hollow blocks, and flat slabs, under seismic actions employing nonlinear dynamic and static analysis. Three-dimensional (3D) finite element models, having different heights selected as 5-, 10- and 15-storey, are developed and analysed using the ETABS structural package^34^. For this purpose, all building models are designed following the requirements of ACI 318-14^35^. In the NDTHA, the developed models are excited using seven earthquake motions from different stations scaled to meet a site-specific hazard. Additionally, the study is extended to expect the level of inelasticity by evaluating the strength reduction factor, R-factor, that indicates the ability of structure to dissipate energy, then comparing calculated values with suggested values by the seismic design code. The rest of the paper is organized as follows. In “Building models” section describes nine RC buildings building models with 5, 10 and 15 stories. In “Earthquake records” section describes a suit of seven-ground motion records with different peak PGAs chosen. In “Nonlinear time history analysis” and “Nonlinear static pushover analysis” sections describe nonlinear time history analysis and nonlinear static pushover analysis. The results discussion is performed in “Results and discussion” Section. Finally, main conclusions are discussed in “Conclusions” Section.

## Building models

**Figure 1:**
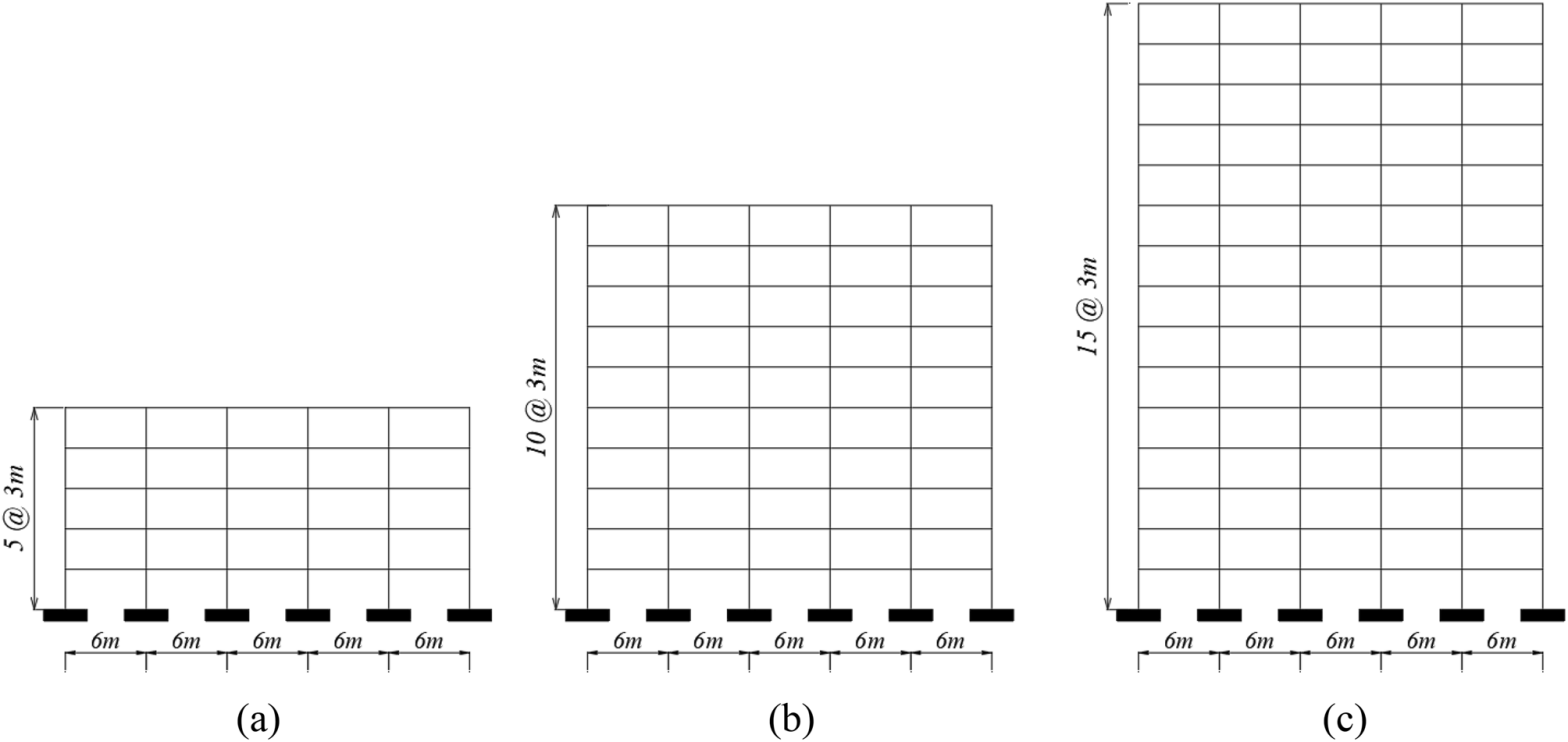
Elevation views of the analysed (**a**) 5-storey, (**b**) 10-storey and (**c**) 15-storey building models.

**Figure 2:**
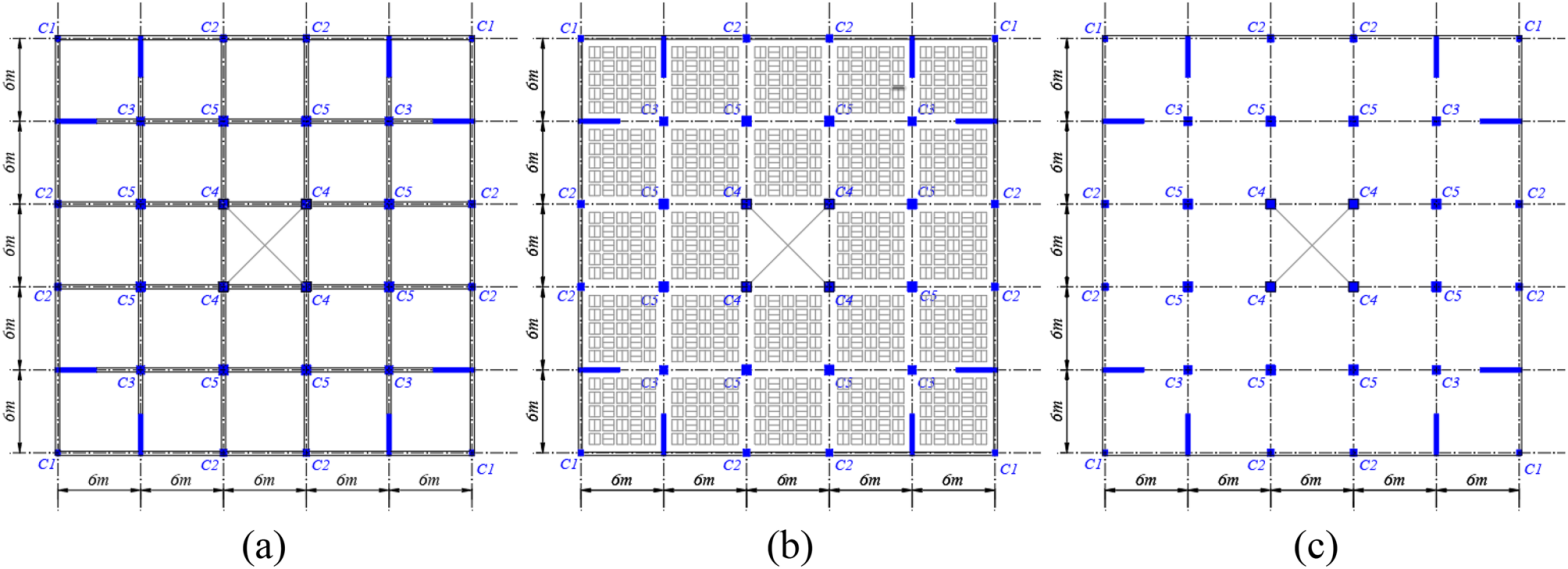
Structural floor plan views showing beams, columns, and shear walls of the building models with various floor systems (**a**) solid, (**b**) hollow block, and (**c**) flat slab systems.

**Figure 3:**
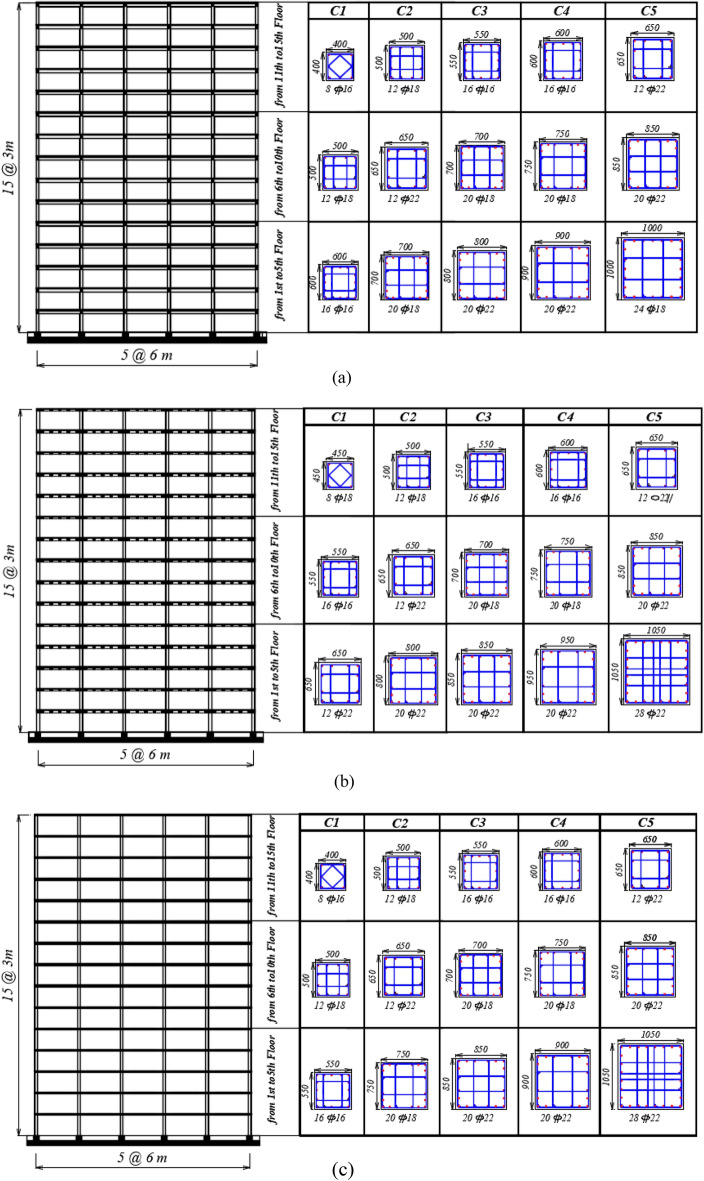
Elevation views of 15-storey building models with columns cross-section dimensions and reinforcement for (**a**) solid, (**b**) hollow block, and (**c**) flat slab systems.

**Figure 4:**
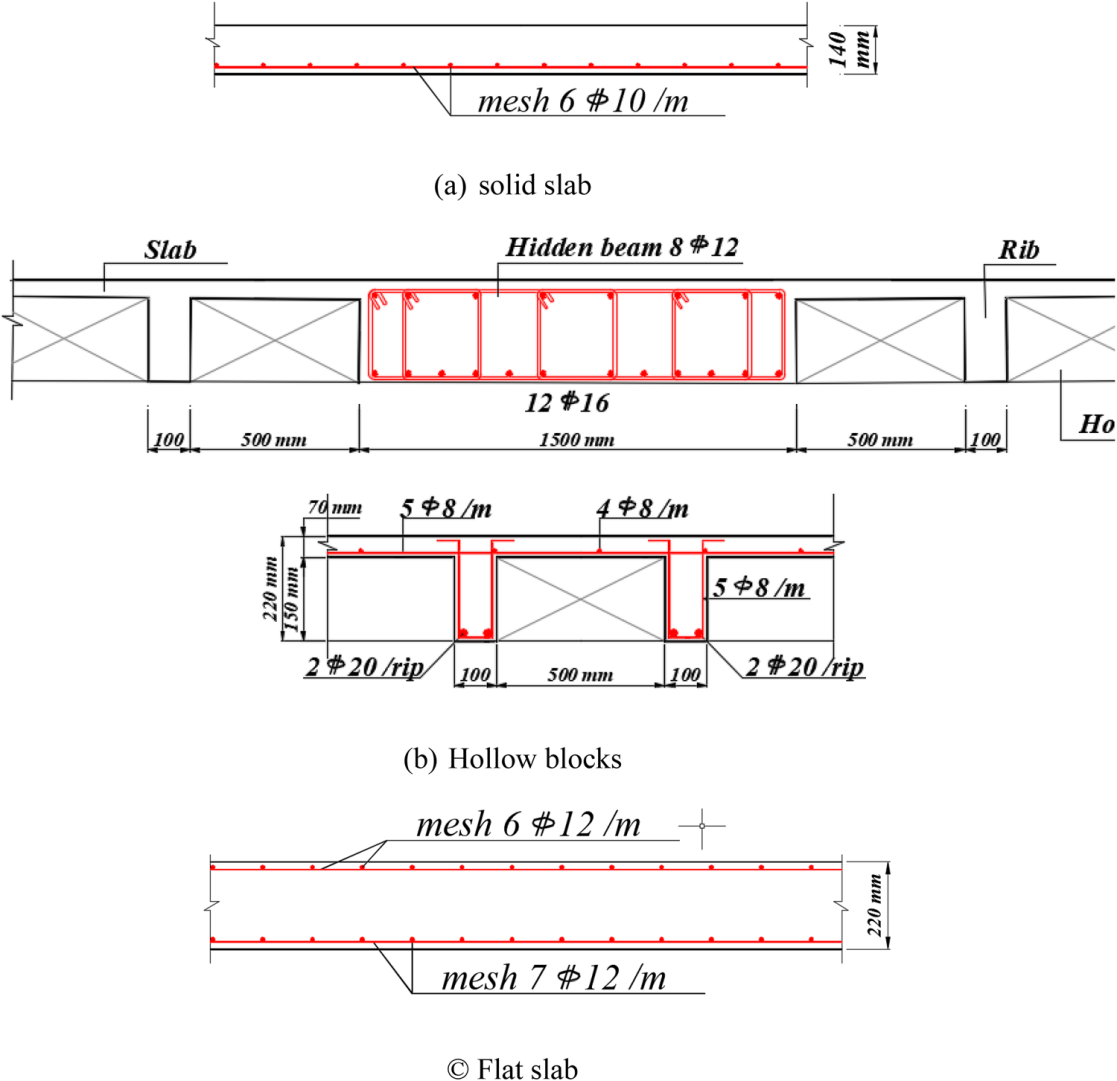
Cross-section details of slabs with steel reinforcement (**a**) solid, (**b**) hollow block, and (**c**) flat slab systems.

In this study, nine RC buildings with 5, 10 and 15 stories (see Fig. 1) are adopted to investigate the seismic behavior of buildings with solid, flat, and hollow block slab systems as illustrated in Fig. 2. The selected buildings to perform the dynamic analysis represent mid-rise buildings^36–39^. The building configurations have a square symmetric floor plane of five spans of equal lengths of 6 m each. The floor-to-floor heights are fixed to be 3 m for all buildings. As a result, the total heights are 15, 30, and 45 m for 5-storey, 10-storey and 15-storey building models, respectively. The seismic design of the selected buildings is conducted following ASCE 7-16^40^ seismic load provisions assuming a seismic zone of peak ground acceleration (PGA) equal to 0.15 g. The associated seismic zone parameters in terms of spectral accelerations at short and 1-s periods are Ss = 0.75 and S_1_ = 0.3, respectively. Medium to stiff soil of type (D) is employed during the simulation analysis. Eight RC shear walls of thickness 300 mm are symmetrically distributed to resist earthquake forces (see Fig. 2). The required flexural and shear reinforcements for horizontal and vertical structural elements are computed according to ACI 318-14^35^. The detailed cross sections of the designed elements are provided in Figs. 3 and 4. The size of the beams supporting solid slabs is 250 mm × 600 mm, while the hidden beams carrying the hollow block system are of dimensions 1000 mm × 250 mm. Based on the previous parameters, three-dimensional models of the buildings were developed utilizing ETABS structural package software, which is considered one of the professional international software used for modelling most of the world-famous towers. Steel reinforcement bars with yield strength of ${f}_{y}=420$ MPa and ultimate strength of ${f}_{u}=520$ Mpa are utilized in designing all structural sections. The concrete compressive strength was assumed to be ${f}_{c}{\prime}=28$ Mpa in calculating section capacities. The utilized modulus of elasticity of concrete is calculated as $4700\sqrt{{f}_{c}{\prime}}$ Mpa. For gravity load design, the values of dead loads, which include the self-weight of structural elements, a typical finishing and partition load, were selected following the design code requirements for residential buildings. A live load of 2 kN/m^2^ is assigned to residential buildings according to ASCE 7-16^40^. Nonlinear finite element analysis in time domain was achieved using ETABS software. To perform the numerical modelling, the slabs and shear walls were modeled using shell elements, whereas the frame elements were utilized to model beams and columns. In order to simulate actual in-plane lateral stiffness, each floor level is considered to be a semi-rigid diaphragm. The auto-meshing option for shell elements was assigned a maximum size of 0.50 m. The buildings were modelled as fixed at foundation level.

The study takes into account the geometric nonlinearity during analysis and design phases that arises as a result of the interplay between axial and transverse forces. The P-Delta analysis feature in ETABS, as a type of geometric nonlinearity, is found on preset P-Delta options where an iterative numerical technique based on applied loads is selected for performing the analysis. A geometric stiffness matrix is formulated based on the obtained member forces from the initial static analysis. The geometric stiffness matrix is repeatedly modified and used to perform subsequent static analyses until the predefined conditions for convergence are satisfied. A full non-linear iterative solution allows all sorts of other non-linear conditions to be accounted for simultaneously, in addition to both the P–Δ and P–δ effects.

**Figure 5:**
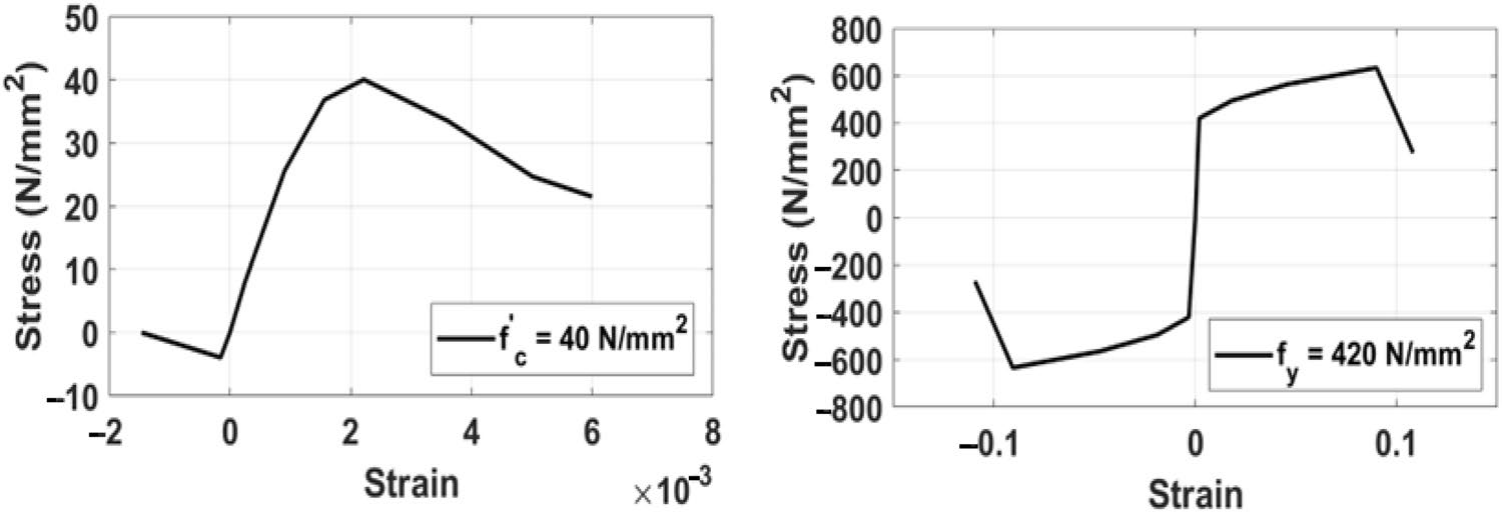
Stress–strain relationship of concrete and reinforcing steel bars.

Shear walls are one of the most effective lateral force-resisting systems that are commonly used in medium- to high-rise buildings where they can provide the needed strength and stiffness for the structure to resist lateral loadings. Shear walls are modelled using elastic elements with nonlinear springs at the top and the bottom of the wall segment. These springs represent concrete and steel fibers along the wall section. The nonlinear analysis requires the stress–strain curves of concrete and steel and the limit states. ETABS is capable of developing the required stress strain curves for concrete and steel provided that the properties of these materials are precisely defined during the input stage. The behavior of the constituent materials of the wall is simulated by the stress-strain relations developed in Fig. 5 for concrete and steel bars in compression and tension. The behavior and status of shell elements like shear walls can be examined by checking out the formation of plastic hinges in the modelled walls.

Depending on the shape, floor system, and number of stories, the dynamic properties of the building will vary. Table 1 exhibits modal time periods and frequencies for nine RC buildings with 5, 10, and 15 stories with solid, flat, and hollow block slab systems.

**Table 1:** Numerically obtained modal time periods and frequencies for building models having a 5% damping ratio.

Building model	Floor system	Natural period (s)		Circular frequency (rad/s)	
		Mode 1	Mode 2	Mode 1	Mode 2
5—storey	Solid slab	0.612	0.533	10.261	11.782
	Hollow block	0.791	0.706	7.939	8.895
	Flat slab	0.865	0.837	7.260	7.503
10—storey	Solid slab	1.351	1.236	4.648	5.081
	Hollow block	1.86	1.645	3.376	3.818
	Flat slab	2.132	1.958	2.946	3.207
15—storey	Solid slab	2.126	2.012	2.954	3.121
	Hollow block	2.976	2.324	2.110	2.702
	Flat slab	3.597	3.108	1.746	2.021

## Earthquake records

**Figure 6:**
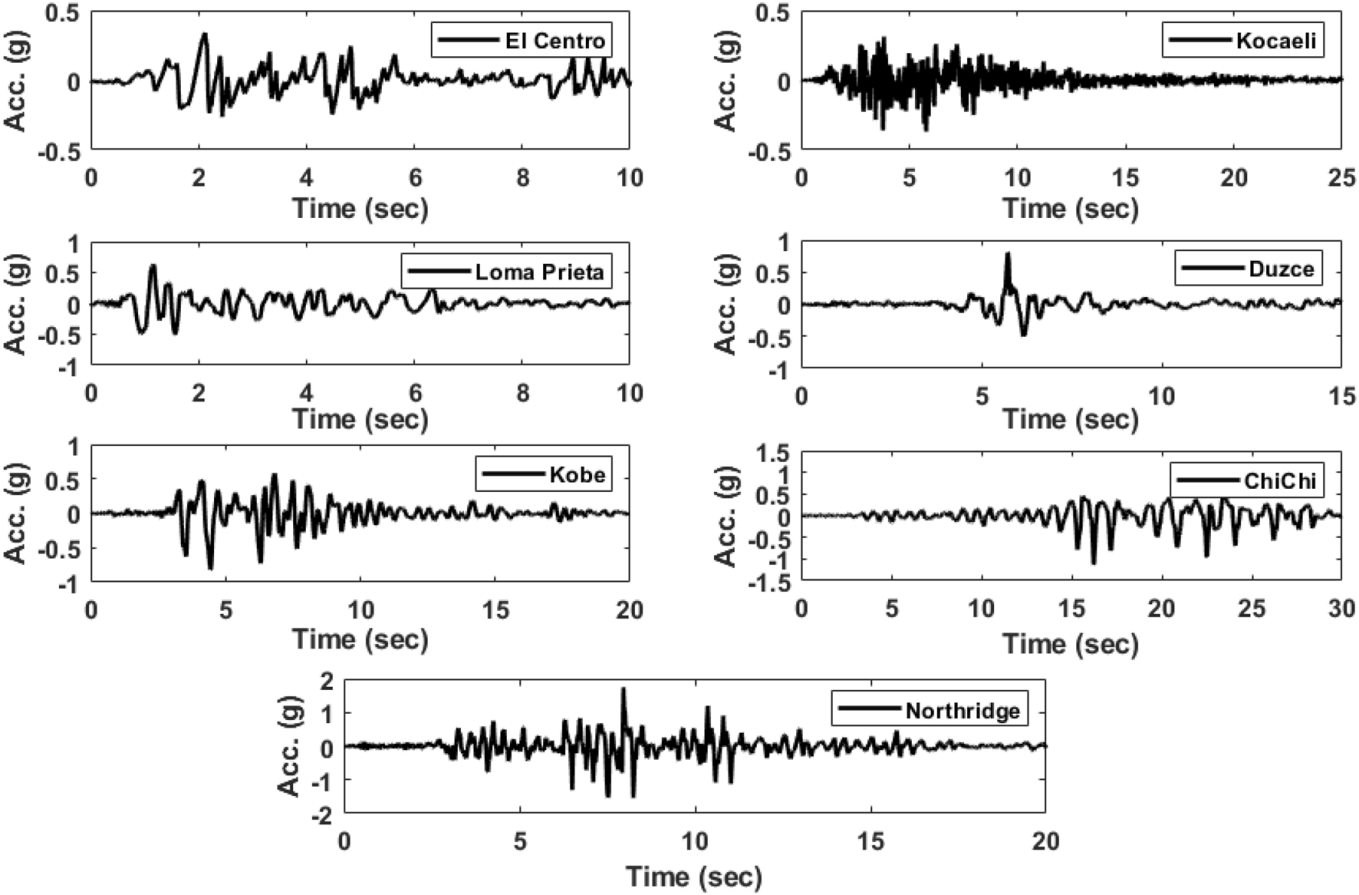
Acceleration time histories for the group of seven earthquake ground motion records adopted in the study.

For performing nonlinear dynamic analysis and evaluating the seismic response of buildings with various flooring systems, a suit of seven-ground motion records with different peak PGAs from different locations is chosen as input excitations. The selected input excitation records were scaled to have the same PGA and match the defined design spectra corresponding to the seismic design code. The real earthquake records are plotted in Fig. 6. In addition, the station, magnitude, PGA, peak frequency content and peak spectral acceleration as key parameters of the selected records are presented in Table 2. It must be emphasized that, according to the ASCE7-16^40^, in the case of choosing seven earthquake records or more to perform the analysis, the average of the calculated response values is acceptable to be used for design purposes. However, if a number of ground motion records less than seven is selected, the obtained peak response value can be considered for performing the design.

**Table 2:** Ground-motion records input to the building models.

Earthquake (record)	Date	Station	Dss (km)	PGA (g)	Mw	ω (m/s)	SA (g)	Soil class	Vs (m/s)
El Centro	1940	117 El Centro	8.30	0.34	7.2	0.32	0.9	D, C	180–760
Kocaeli (Izmit)	1999	Sakarya	3.10	0.37	7.4	0.11	2.5	B, B	
Loma Prieta	1989	14 WAHO	16.9	0.63	6.9	0.75	1.1	D, –	
Duzce	1999	375 Lamont Station	26.0	0.97	7.1	0.36	1.4	D, D	
Kobe	1995	KJMA	0.60	0.82	6.9	0.58	2.1	B, B	
Chi–Chi	1999	TCU129	1.18	1.14	7.6	1.00	3.4	–, C	
Northridge	1994	74 Sylmar	6.20	1.75	6.7	0.89	4.2	D, C	

*PGA* peak ground acceleration, *Mw* magnitude, *ω* peak frequency content of the earthquake, *SA* peak spectral acceleration of the earthquake, *Dss* site-source distance, *Vs* shear wave velocity.

**Figure 7:**
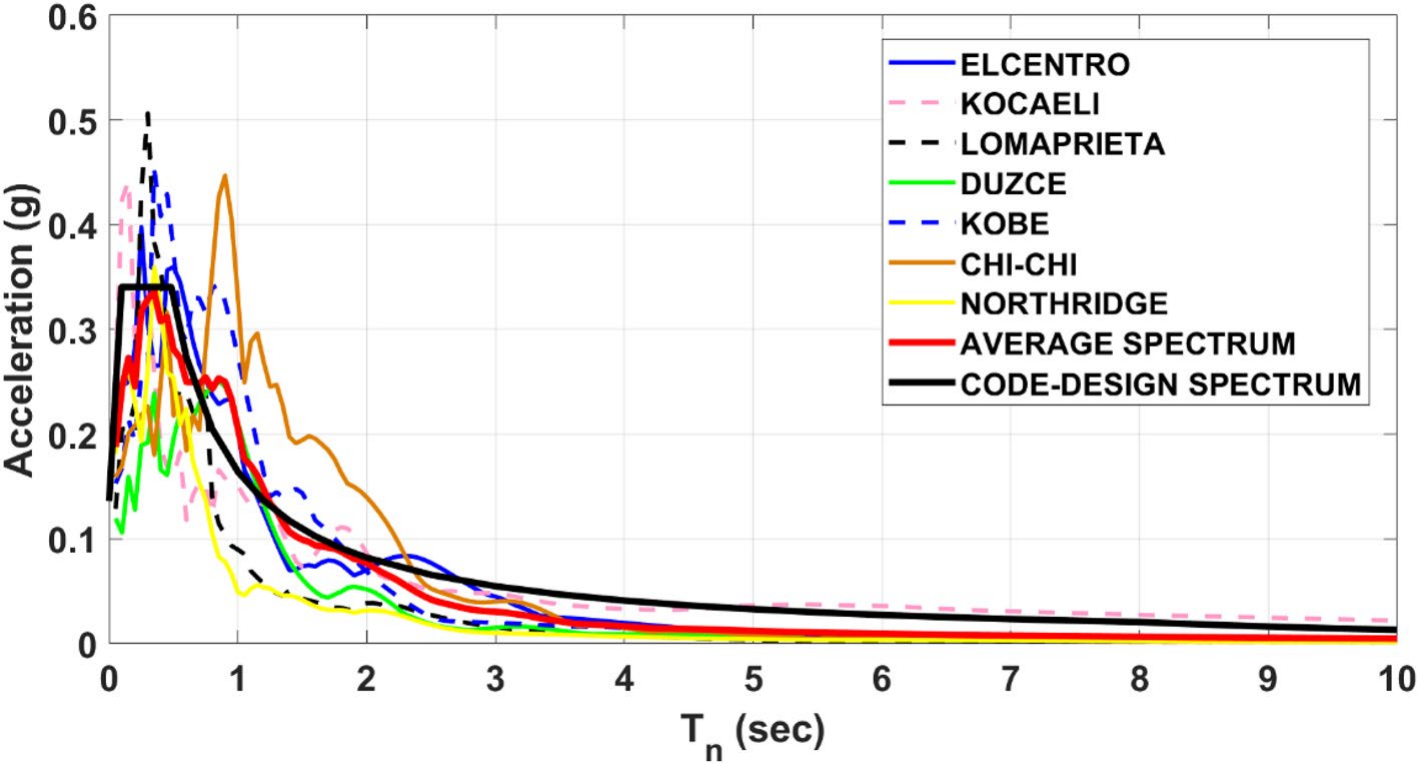
Elastic and average spectrum for the selected earthquake records against the demand spectrum curve provided by ASCE 7–16^40^.

Several techniques are available for performing the scaling of earthquake excitation records following the seismic design code requirements^41–43^. The amplitudes of the selected records are scaled such that the median spectrum of the spectra of the scaled ground motion records matches the target spectrum of the considered seismic hazard intensity (see Fig. 7).

**Figure 8:**
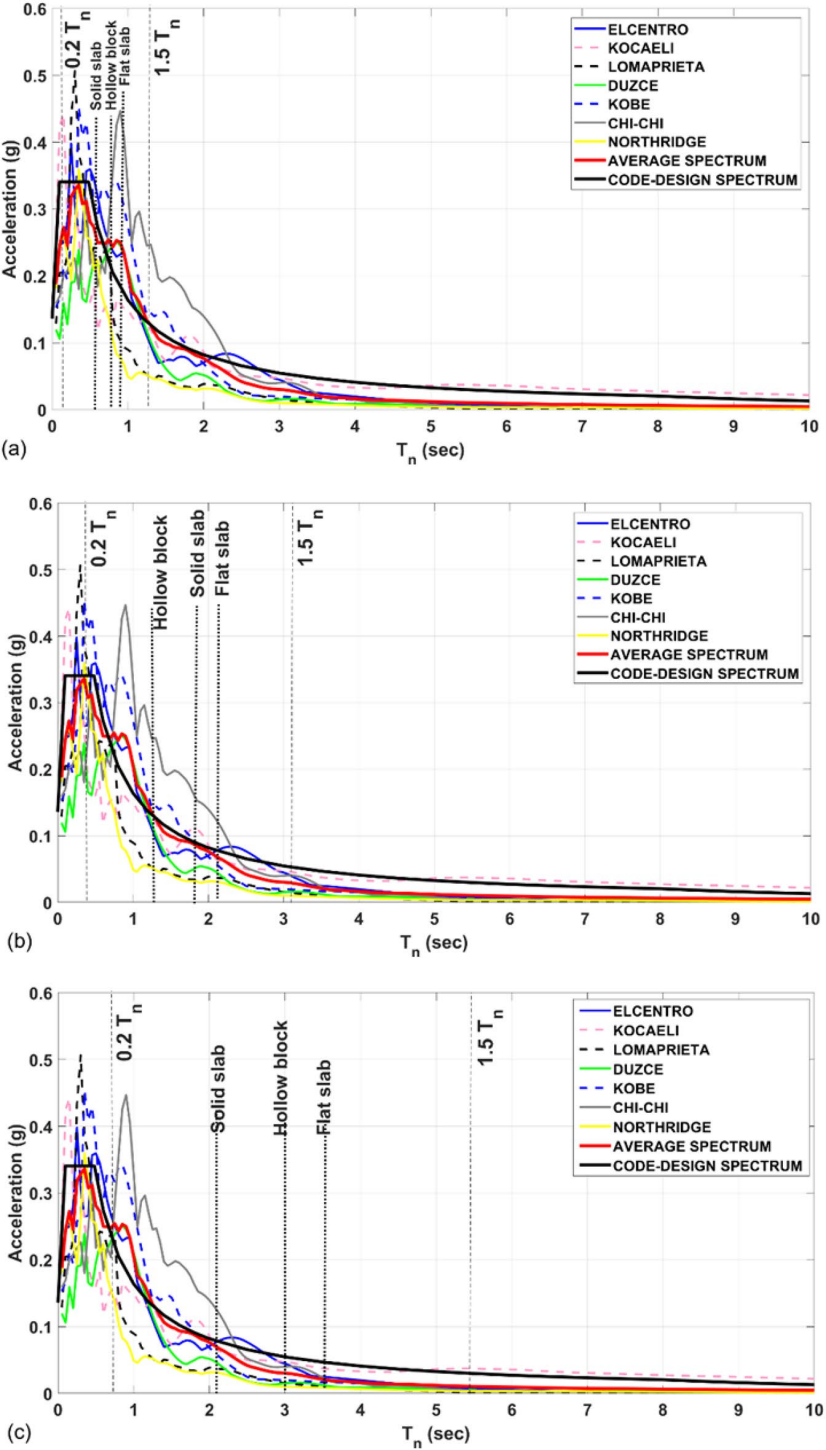
Elastic and average spectrum for the selected earthquake records against the demand spectrum curve provided by ASCE 7–10 for (**a**) low-rise buildings, (**b**) mid-rise buildings and (**c**) high-rise buildings.

For scaling purposes, the amplitudes of the selected records are scaled such that the median spectrum of the spectra of the scaled ground motion records, matches the target spectrum of the considered seismic hazard intensity within the period range of 0.2 T and 1.5 T following the design, where T is the fundamental period of the building. The range of interest as per the requirements of ASCE 7-10 for seismic response history procedures is considered as varying from 0.2 to 1.5 T. The plotted spectrum of each of the selected earthquake records matches 5% damped ASCE-7-10 targeted design spectrum seeking minimum error in the range of interest. For research purpose, the authors records are selected to provide mean spectrum that minimizes the induced error with the deign targeted spectrum particularly in the range of interest 0.2–1.5 T (see Fig. 8).

## Nonlinear time history analysis

Time history analysis is addressed in almost all seismic codes as the most reliable technique. This can be due to its capability to model a wide range of nonlinear material behaviors and geometric nonlinearities. Also, time history analysis can reflect the nonlinear changes in structural stiffness under seismic actions. However, it also has several disadvantages, including extremely time-consuming analysis, more efforts to develop effective analytical models, and sensitivity to system parameters. In the current study, NDTHA is performed under the action of different ground motion records scaled to fit the region of study. In this method, the dynamic motion equation is numerically integrated to get the seismic response of buildings exposed to a specified earthquake load at discrete time intervals employing the iteration technique. Meanwhile, NDTHA requires iterations particularly when nonlinear dynamic behavior is developed in the structure and the actual stiffness of the complete structural system needs to be recalculated due to degradation of strength as well as redistribution of forces. The dynamic motion equation for multistory buildings in the elastic behavior stage can be written as^44^:

1 $$[M]\left\{\ddot{u}\right\}+[C]\left\{\dot{u}\right\}+[K]\left\{u\right\}=-[M]\left\{I\right\}{\ddot{u}}_{g}$$

where ${\varvec{M}}$, ${\varvec{C}}$ and ${\varvec{K}}$ respectively refer to mass, structural damping, and stiffness matrices, as illustrated Eqs. (1a)–(1c) and $U$ is defined as the response vector for the structure:

1a $$\left[{\varvec{M}}\right]=\left[\begin{array}{ccccc}{m}_{1}& 0& \cdots & 0& 0\\ 0& {m}_{2}& \cdots & 0& 0\\ \vdots & \vdots & \ddots & \vdots & \vdots \\ 0& 0& \cdots & {m}_{n-1}& 0\\ 0& 0& \cdots & 0& {m}_{n}\end{array}\right]$$

1b $$\left[{\varvec{C}}\right]=\left[\begin{array}{ccccc}{c}_{1}+{c}_{2}& -{c}_{2}& \cdots & 0& 0\\ -{c}_{2}& {c}_{2}+{c}_{3}& \cdots & 0& 0\\ \vdots & \vdots & \ddots & \vdots & \vdots \\ 0& 0& \cdots & {c}_{n-1}+{c}_{n}& -{c}_{n}\\ 0& 0& \cdots & -{c}_{n}& {c}_{n}\end{array}\right]$$

1c $$\left[{\varvec{K}}\right]=\left[\begin{array}{ccccc}{k}_{1}+{k}_{2}& -{k}_{2}& \cdots & 0& 0\\ -{k}_{2}& {k}_{2}+{k}_{3}& \cdots & 0& 0\\ \vdots & \vdots & \ddots & \vdots & \vdots \\ 0& 0& \cdots & {k}_{n-1}+{k}_{n}& -{k}_{n}\\ 0& 0& \cdots & -{k}_{n}& {k}_{n}\end{array}\right]$$

1d $$\left[U\right]=\left[{u}_{1}{u}_{2}\cdots {u}_{n-1}{u}_{n}\right]$$

The vector $I$ represents a unit vector and ${\ddot{u}}_{g}$ refers to the ground acceleration records.

The Rayleigh damping model, which is the most common—almost widespread for viscous damping in nonlinear response history analysis of buildings, is used to create damping forces in the considered building models. The damping matrix can be written as a linear combination of the mass and stiffness matrices. This combination is written as $c={a}_{0}m+{a}_{1}k$, where the coefficients ${a}_{0}$ and ${a}_{1}$ are determined from damping ratios specified in either two natural modes of vibration of the structure or at two selected frequencies.

For inelastic behavior stage, the dynamic equation of motion can be expressed as^45^:

2 $$[M]\left\{\ddot{u}\right\}+[C]\left\{\dot{u}\right\}+\left\{\mathrm{fy}\right\}=-[M]\left\{I\right\}{\ddot{u}}_{g}$$

where ${f}_{y}$ is the yield force vector. The Newmark β method is used for the time integration of the dynamic equilibrium equations of motion at each time step.

## Nonlinear static pushover analysis

Currently, POA is being widely used as one of the fastest nonlinear analyses. POA, as a nonlinear static analysis method, is characterized by the rapid estimation of the inelastic structure responses due to its inherent simplicity in modeling and saving computational time. The use of NDTHA in assessing the seismic performance of structures requires the use of specific numbers of ground motion records, according to the recommendations of the design code, to determine the ratio of demand-capacity (D/C). On the other hand, POA can serve as an alternative procedure to the comprehensive NDA by using the earthquake design spectrum curve together with the capacity curve to estimate the average peak of displacements. The D/C ratio, for performance assessment, is calculated based on these captured displacement values. In addition, POA enables the user to estimate the load-bearing capacity, global displacement ductility, stiffness, and failure mechanism of a structure under the applied seismic loads following the application of gravity loads. The seismic load is increased gradually to enable monitoring of the progressive yielding, cracking, and stiffness degradation of structural elements. This process is continued by applying a step-by-step displacement-controlled technique until the structure becomes unstable or reaches the predetermined level of maximum lateral displacement. One of the most important outputs of POA is the force–displacement relationship. The three key steps for performing POA are: (1) development of the pushover curve; (2) estimation of the target nodal displacement; and (3) checking that the safety functions fulfill the corresponding performance level. It is worth noting that the conventional POA is appropriate for buildings whose dynamic behaviour is solely dependent on a single-mode response, such as most low-rise and medium-rise regular systems. Therefore, applying this method to high-rise buildings or asymmetric-plan buildings may not be acceptable and produce inaccurate results. Enhanced pushover processes have been developed to overcome this restriction. A number of different approaches to incorporate the higher mode effects such as modal pushover analysis (MPA)^46^, generalized pushover analysis^47^, consecutive modal pushover analysis (CMP)^48^, extended N2 method^49^, adaptive pushover analysis^50^, spectrum-based pushover analysis (SPA)^51^, and multimode pushover analysis^52^, have been developed.

### Target displacement

**Figure 9:**
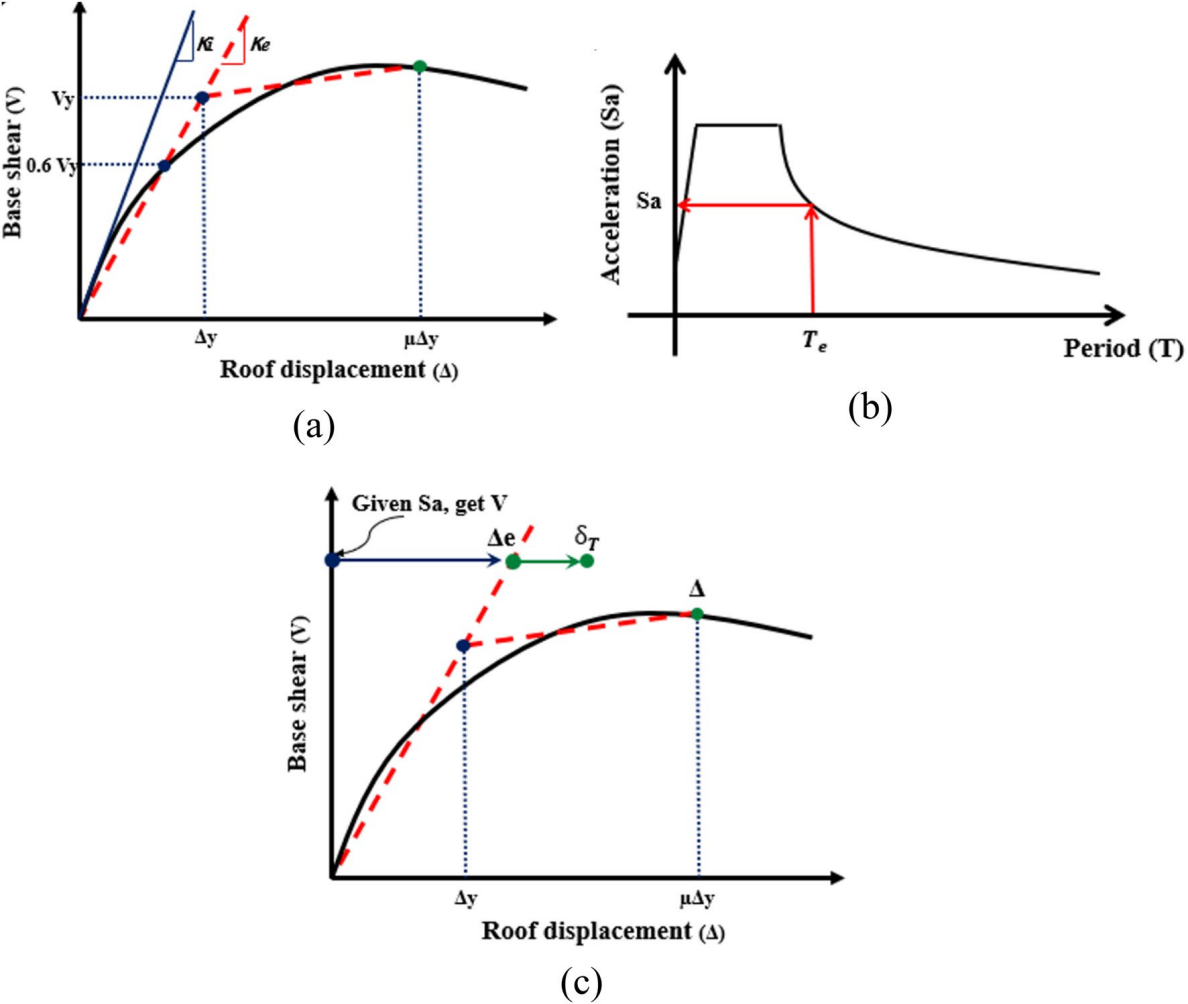
Main steps of FEMA 440 method.

The target displacement represents the damage experienced by a building subjected to an earthquake excitation. Several procedures can be utilized to estimate the target displacement, namely displacement coefficient method (DCM), the capacity spectrum method (CSM), the improved coefficient method (ICM), and the improved capacity spectrum method (ICSM). The procedures of these methods are respectively available and specified in FEMA-356^53^, ATC-40 report^54^, ASCE-41^55^, and FEMA-440^56^. A schematic overview of the assessment of target displacement following the procedures provided in FEMA 440^56^ is explained in Fig. 9. Once the pushover curve is determined, one may convert it into a bilinear force–deformation relationship such that the area under the approximated curve is almost equivalent to the original one. The formation of the bilinear curve provides both yield force ${V}_{y}$ and effective stiffness ${K}_{e}$. The effective natural period of the building ${T}_{e}$ can be calculated in terms of the initial stiffness ${K}_{i}$ and natural period ${T}_{i}$ and ${K}_{e}$ as

3 $${T}_{e}={T}_{i}\sqrt{\frac{{K}_{i}}{{K}_{e}}}$$

The corresponding spectral acceleration ${S}_{a}$ to the calculated ${T}_{e}$ can be assigned as indicated in the provided Fig. 3b. Similarly, the corresponding base shear value $V$ to ${S}_{a}$ is assigned and consequently the elastic displacement ${\Delta }_{e}$ is determined as well. Scaling such obtained elastic displacement yields the target displacement ${\delta }_{T}$ as:

$${\delta }_{T}={C}_{0}{C}_{1}{C}_{2}{C}_{3}{S}_{a}\frac{{T}_{e}^{2}}{4{\pi }^{2}}$$

The constants ${C}_{0}$, ${C}_{1}$, ${C}_{2}$ and ${C}_{3}$ are modification factors defined as in Table 3.

**Table 3:** Definitions of constants ${C}_{0}$, ${C}_{1}$, ${C}_{2}$ and ${C}_{3}$.

Constant	Definition
${C}_{0}$	Modification factor to relate the elastic displacement of SDOF system to MDOF one
${C}_{1}$	Modification factor to relate the maximum inelastic and elastic displacements of SDOF system
${C}_{2}$	Modification factor to represent the effect of hysteretic shape, stiffness, and strength degradations
${C}_{3}$	Modification factor to represent the increments in displacements due to $P-\Delta$ effects

### Nonlinear behavior of the proposed models

**Figure 10:**
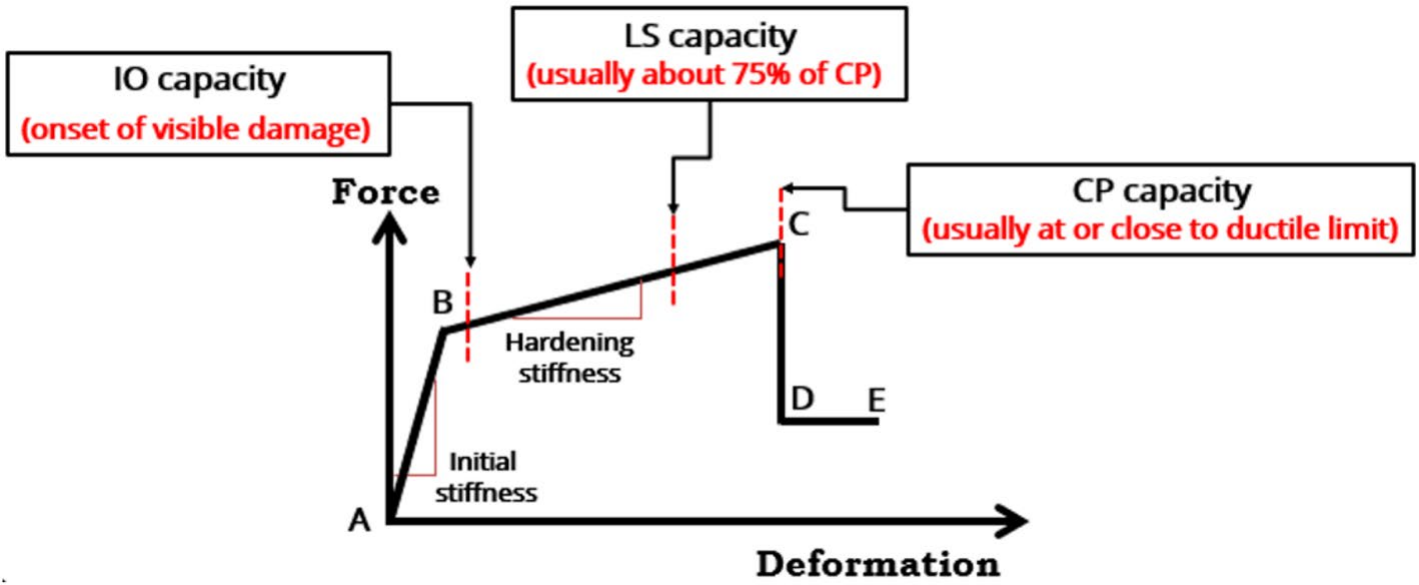
Acceptance criteria by FEMA-356 (IO, LS, and CP) for force–deformation relation of plastic hinge.

Inel and Ozmen^57^ performed a study to investigate the possible differences between pushover analyses of the default-hinge and user-defined hinge models. Although the study indicated that implementing a user-defined hinge is better than the use of default-hinge one in reflecting nonlinear behavior compatible with element properties, the default-hinge model, provided in ETABS, can be used for beams and columns with careful attention to avoid the misuse of the default-hinge properties. In pushover analysis procedure, the structural members are modeled to behave nonlinearly by defining the default plastic hinges (i.e., a lumped plasticity model) at different locations in frame, commonly located at ends of the frame element. Default nonlinear hinge properties are adopted as ASCE 41-13. Flexural moment hinges (M3) are assigned to beams, while coupled bending moment and axial force (P-M3) are assigned to columns. Moment and rotation behavior of plastic hinges is usually characterized by five points labeled (A, B, C, D, and E) as presented in Fig. 8. Assigning the values to each of these points may change according to the type of element, material properties, the level of axial loads*,* and the ratio of longitudinal and transverse reinforcement. As shown in Fig. 10, the linear behavior is represented between the origin point at A and the yield point at B. A small percentage of slope, 0–10% of the elastic slope, in the strain hardening zone from yield point B to ultimate point C. An abscissa value corresponding to point C represents the deformation at the beginning of major strength degradation (see line CD). After point D, the component responds with continues reduced strength to point E. The typical rotation values corresponding to each point on the graph is also shown in the same figure. Immediate occupancy (IO), life safety (LS) and collapse prevention (CP) are the three points utilized to identify the performance acceptance criteria for the hinge. FEMA-356 pre-standard established the acceptance criteria and recommended parameter values for modeling RC beams and columns.

## Results and discussion

### Results of time history analyses

#### The fundamental periods

As one of the most important dynamic characteristic parameters that control the seismic demand under the action of lateral loads, the fundamental period of vibration of a structure, which is obtained according to eigenvalue analyses and is denoted by the parameter Tn, enables the designer to gain insight into the behavior of the structure when it is subjected to seismic activity. Moreover, it plays a significant role in the design of new buildings and the risk assessment of existing ones as well. Seismic design codes provide approximate formulas for estimating the natural period of vibration in terms of the structural system, height, and type of construction materials^58^.

**Figure 11:**
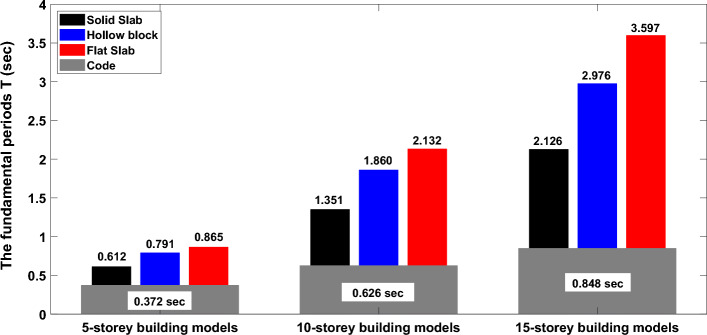
Values of the fundamental natural periods of 5-storey, 10-storey, and 15-storey building models with various floor systems.

Following the provided Table 12.8-2 in the ACI-7-16 Code, the ${T}_{n}$ values for 5-, 10-, and 15-story RC buildings with different slab systems are compared in Fig. 11 against ${T}_{n}$ values based on eigenvalue analysis and Rayleigh method considering cracking structural elements. The figure shows that the estimated values following code of design for all building models and floor systems are lower than those obtained from the dynamic analysis employing the lateral stiffness and seismic masses of the cracked structural elements. The underestimation of the code-based ${T}_{n}$ values of structures may result in the overestimation of the seismic demands. The captured ${T}_{n}$ values for the considered building models with various heights and floor systems indicate that the lateral stiffness of covering floor slab may significantly alter the natural period of structures having same height. The building models with solid slabs show the lowest natural period as compared to building models with hollow block or flat slab. That is attributed to the increase of floor stiffness as a result of existence of drop beams. In addition, the presented figure clearly indicates that as building heights increase, the influence of floor system on the induced natural periods becomes more pronounced and noticeable. More specifically, for low-rise building models, the hollow block and flat slabs provide, respectively, about 29% and 41% increase in the obtained time periods compared with the time period of solid slab system. Likewise, the captured fundamental time periods for hollow block and flat slabs in 10-storey buildings offer about 38% and 58% percentages of increment, respectively. The corresponding percentages of increase of fundamental periods of 15-storey building models are 40% and 69%, respectively. Results from the performed analysis clearly indicate that building models with flat slab as a flooring system provide the highest ${T}_{n}$ values while ones with solid slabs provide the lowest values of ${T}_{n}$ leading to the fact that the floor system substantially minimizes or maximizes the induced fundamental periods of structures.

#### Base shear coefficients

**Figure 12:**
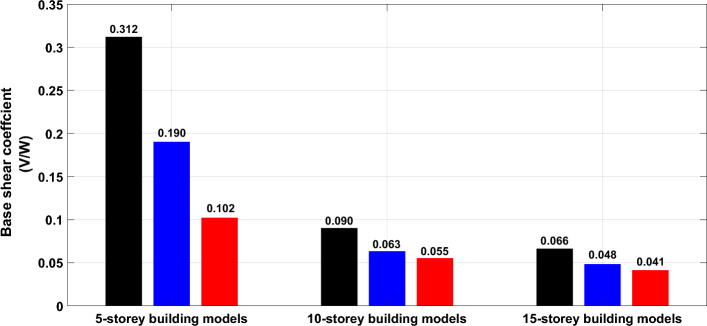
Base shear coefficient of the designed buildings.

Figure 12 presents the average base shear coefficients for all the considered structural models of different numbers of stories and floor systems employing the seven scaled time-history records. The base shear coefficient refers to the maximum design base shear normalized by the structure’s weight and is commonly used in controlling the structures seismic performance. The bar graph reported in the figure confirms that the base shear coefficients significantly decreased according to the increase in height of the building. Regardless of the building’s height, flat slab system shows the lower base shear coefficient compared to the hollow block and solid slab systems. Additionally, the solid slab system has higher base shear coefficients compared with other slab systems.

It is observed from the figure that the differences among base shear coefficients corresponding to different slab systems are highly pronounced for building models of low heights. Quantitively, the average base shear coefficients for the case of building models of low heights obtained for three systems, solid, hollow block and flat slabs are: 0.312, 0.190 and 0.102, respectively; while the corresponding values for the case of 10-storey building models are: 0.090, 0.063 and 0.055, respectively. For high-rise building models, the values of base shear coefficients are: 0.066, 0.048 and 0.041, respectively. This means that the buildings with adopted hollow blocks and flat slab systems being lighter and more flexible, accordingly absorb smaller seismic shear forces in comparison to the same building with adopted solid slab system. Since the design of earthquake-resistant structures is highly controlled by the shear at the base, changing the slab type can substantially influence the design methodology.

#### Displacement profiles

Imposed seismic load on structures during an earthquake may result in weaker and more flexible buildings with higher displacement values. The ASCE-7-10 seismic design code requires that the designer assess the effects of lateral displacements on both structural and non-structural elements to prevent the detrimental effects of inelastic deformations of the primary structure. It is worth noting that, without judicious building designs, the expected lateral deformation becomes too large, exceeding the prescribed distances between adjacent structures, leading to pounding of adjacent buildings against each other during an earthquake event. In addition, if the induced lateral displacements become too large, the structure can develop P–∆ instability and might suffer premature damage that may result in potentially collapse as well. Consequently, it is important to provide adequate stiffness to avoid excessive displacements.

**Figure 13:**
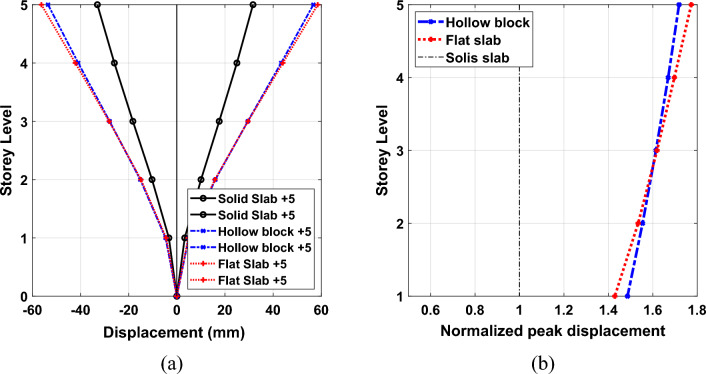
Comparison of the (**a**) average peak displacement patterns and (**b**) normalized peak displacement for 5-story building models exposed the designated ground motion records.

**Figure 14:**
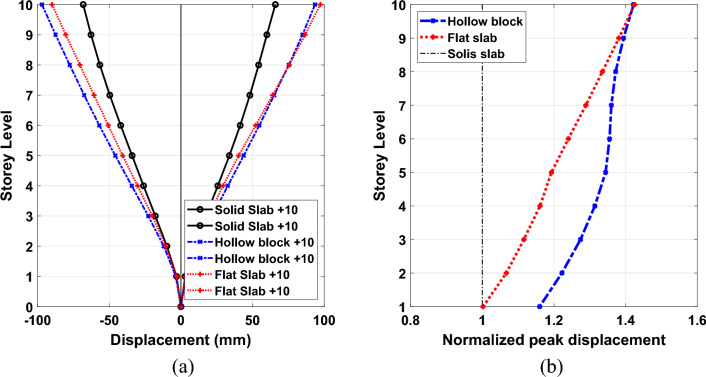
Comparison of the (**a**) average peak displacement patterns and (**b**) normalized peak displacement for 10-story building models exposed the designated ground motion records.

**Figure 15:**
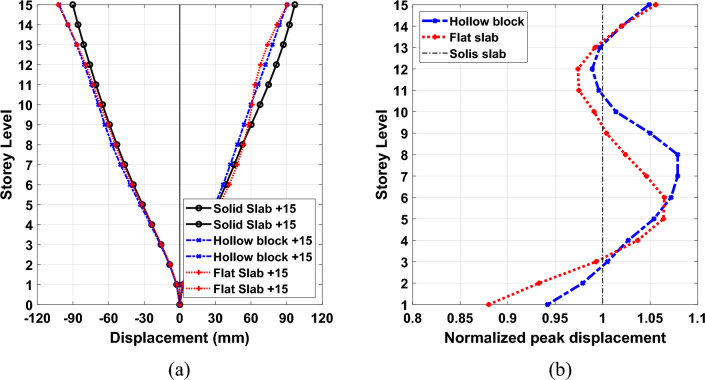
Comparison of the (**a**) average peak displacement patterns and (**b**) normalized peak displacement for 15-story building models exposed the designated ground motion records.

The presented herein is the height-wise distribution of average peak displacement response demand under the considered seven ground motion records. The normalized peak displacement values at each storey level to the corresponding responses at each story of solid slab system are presented as well in Figs. 13, 14 and 15 for 5-storey, 10-storey and 15-story building models, respectively. As it is expected, the displacement plots show that the maximum displacements are captured at the top floor of all the different types of building onsidereds. The figure illustrates that the calculated average peak story displacements increase in an almost linear trend along the story levels for all the considered building models. In addition, the stiffest solid slab system gives the smallest values of displacement compared with flexible hollow blocks and flat slabs. Such an increase in lateral story displacements of the other two systems can be attributed to the overall reduction in global transverse stiffness resulting from the absence of beams which can form strong frame actions with columns. The effect of floor slab types on the induced seismic displacements are more pronounced in buildings of 5 and 10 storeys, (see Figs. 13 and 14). The plotted results clearly reveal that the solid slab building models provide lateral displacement profiles smaller than the counterparts of hollow black and flat slab building models particularly for the 5-story and 10-story models. Quantitively, the captured peak floor displacements for building models of 5 storeys are 31.63 mm, 56.70 mm and 58.51 mm for systems of solid, hollow block, and flat slabs, respectively. For 10-storey building models, the corresponding peak displacement values are 65.86 mm, 93.54 mm and 97.20 mm for systems of solid, hollow block, and flat slabs, respectively. In addition, the highest values of the roof displacement for the referred slab systems increased to 96.94 mm, 101.86 mm and 102.41 mm for high-rise building models, respectively. From percentage point of view, the obtained roof displacement of the 15-story solid slab building model was reduced by about 5% compared with the other two systems. The corresponding percentages of reduction for peak displacements of 10-story and 5-story solid slab building models were found of about 32% and 45% compared with the other two floor systems. It is worth noting that, the hollow block and flat slab systems give roughly close trend of the induced displacement profile along the building height.

#### Drift profiles

**Figure 16:**
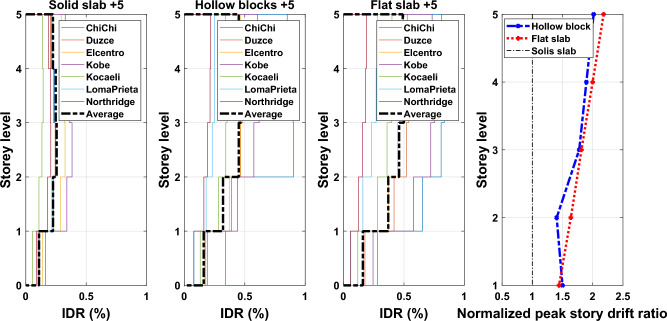
Comparison of the inter-story drift ratios and the normalized peak story drift ratios for the 5-story building models exposed the designated ground motion records.

**Figure 17:**
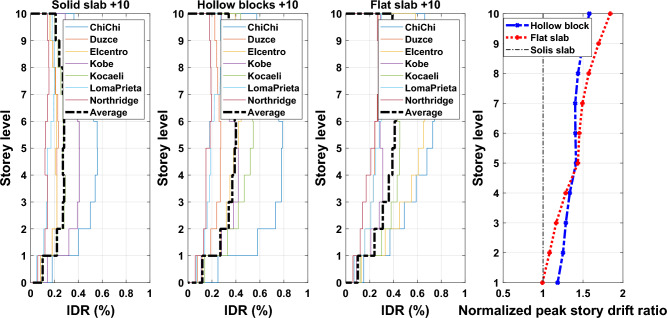
Comparison of the inter-story drift ratios and the normalized peak story drift ratios for the 10-story building models exposed to the selected ground motion records.

**Figure 18:**
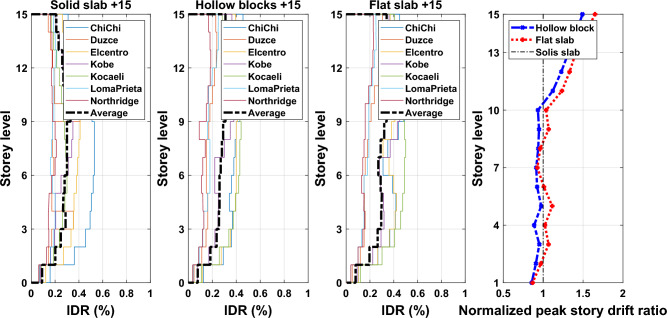
Comparison of the inter-story drift ratios and the normalized peak story drift ratios for the 15-story building models exposed to the selected ground motion records.

Inter-story drift ratio (IDR) is a significant demand indicator of structural performance particularly for structures designed to exhibit nonlinear responses. IDR can be described as the relative drift of successive stories divided by the height of that story. The IDR is utilized to assess the seismic performance of existing structures in displacement-based method, and is also used to evaluate the marginal satisfaction of serviceability limits in a force-based seismic design strategy for new structures. Also, peak IDR generated by earthquakes can be used to evaluate the damage of non-structural elements such as pipework, partition cladding, and ceilings. Therefore, reducing the seismic vulnerabilities of non-structural components requires limiting the ratio to an appropriate value. The average response quantities of IDR profiles obtained from nonlinear time history analyses along the building height for the considered different ground motion records of the adopted buildings are illustrated in Figs. 16, 17, and 18 for 5-, 10- and 15-story building models. The plotted curves obviously show that the obtained IDR demands of 5-, 10- and 15-storey have significant changes. The captured peak IDR values for 5-storey building with systems of solid, hollow block and flat slabs were 0.26%, 0.47% and 0.5%, respectively. The corresponding peak values for 10-storey and 15-storey building models are: 0.29%, 0.41%, 0.42% and 0.3%, 0.38%, 0.41%, respectively. It can be concluded from the results that the maximum IDRs are influenced by the lateral stiffness of floor slab systems where buildings with hollow block and flat slab systems sustain extreme IDR values. Moreover, the distribution of the average IDRs was also influenced by the adoption of floor slab systems, as can be seen in the presented figures. Therefore, an appropriate consideration should be taken to avoid induced excessive IDRs during the design process. Otherwise, adverse effects on non-structural elements as well as the designed structural elements that are part of the lateral force-resisting system may occur.

### Results of pushover analysis

#### Capacity curves for building models

**Figure 19:**
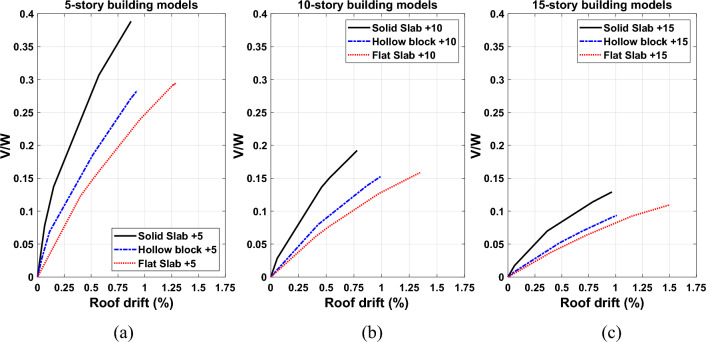
Pushover curves with normalized force–displacement relationships for the (**a**) 5-storey (**b**) 10-storey and (**c**) 15-storey RC frame buildings.

Figure 19 shows comparison plots of typical capacity curves that relate shear force at the base and the roof drift ratios (RDRs) for the 5-, 10- and 15-stories building models with different slab systems. The base shear force is normalized by seismic weight of the structure. From the plotted curves, it can be observed that the normalized base shear values decrease with the building height increment. The captured percentages of peak RDRs for 5-storey building models at the end of pushover analysis are 0.87, 0.93 and 1.29 for the solid, hollow block and flat slabs, respectively. RDRs values are 0.78, 1.0 and 1.35 for 10-storey building models and 0.97, 1.01 and 1.5 for 15-storey building models, respectively. Numerical values clearly reveal that the building model with a solid slab system creates a highly remarkable increase in lateral load, initial-stiffness and deformation capacities compared with the other covering systems. Instead, the models of buildings with hollow blocks or flat slabs show a loss of strength with excessive failure. The results show that the hollow block and flat slabs covering the 5-storey building models increase the value of RDRs by about 7% and 48% compared with the RDRs obtained using the solid slab system, respectively. The corresponding percentages of increase of RDRs are 22%, 73% and 4%, 55% for 10-storey and 15-storey building models, respectively.

**Figure 20:**
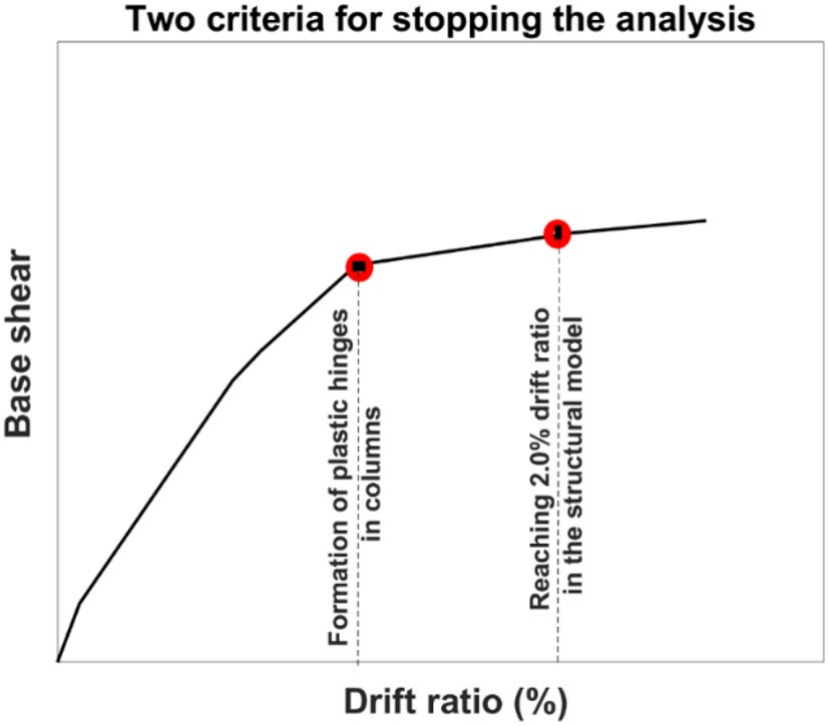
Criteria for stopping the nonlinear static analysis.

During pushover analysis, the seismic load is increased gradually to enable monitoring of the progressive yielding, cracking, and stiffness degradation of structural elements, thereby capturing the formation of plastic hinges in beams and columns throughout the structure. This process is continued by applying a step-by-step displacement-controlled technique until the structure becomes unstable, i.e., forms plastic hinges in columns, or reaches the predetermined level of maximum lateral displacement (see Fig. 20). Whichever happens faster, the analysis is stopped. The maximum lateral displacement level is attained when the maximum story drift ratio reaches 2.0% based on the life safety performance criterion proposed by FEMA. It should be noted that, in the case of formation of plastic hinges in columns, even if the target story displacement limit is not reached, the analyses are terminated. Based on the results, the criteria for stopping the analysis was the formation of the plastic hinges in the supporting columns, since it happened earlier than reaching the predetermined level of maximum lateral displacement.

#### Roof displacement demands

In order to understand the impact of slab systems on the displacement response of structures, the maximum roof displacement demand of nonlinear response time history analyses for a set of real recorded earthquake ground motions and the inelastic demand spectrum by using the results of pushover analysis are presented in Table 4. The table obviously indicates large variation in lateral displacement demands for the nonlinear time history analysis of the selected earthquake records; the ratios of the highest and lowest seismic inelastic displacement demands range from 3.03 to 7.14*.* For a more accurate evaluation, the average seismic demand of ground*-*motion records is also provided in Table 3. The results show that the inelastic displacement demands from nonlinear response time history analyses and nonlinear static analyses are significantly different for all types of models used for the analysis*.* This is evident in the case of 10-storey to 15-storey buildings*.* Moreover, the results clearly indicate large variation in inelastic displacement demands depending on ground motion record, slab type, and static and dynamic analysis of the structure. The displacement demands tend to increase for flat slab building models, especially for 5-storey and 10-storey buildings.

**Table 4:** Roof displacement demands of the building models.

Record	Roof displacement demands of the building models (mm)								
	5-storey building models			10-storey building models			15-storey building models		
	Solid slab	Hollow block	Flat slab	Solid slab	Hollow block	Flat slab	Solid slab	Hollow block	Flat slab
ChiChi	36.71	112.43	101.45	132.78	204.18	171.2	178.33	135.79	119.18
Duzce	29.94	58.00	65.38	58.97	72.20	59.49	66.13	61.55	65.10
Elcentro	46.24	57.97	70.12	48.66	99.19	153.9	138.92	154.92	125.01
Kobe	51.51	72.80	90.81	113.77	91.90	83.04	90.68	96.08	99.48
Kocaeli	16.98	42.63	45.79	65.02	133.88	106.7	109.05	231.45	298.95
LomaPrieta	30.48	30.80	32.38	39.20	49.54	65.98	60.14	54.16	54.94
Northridge	25.54	32.43	30.21	32.03	39.57	56.28	47.18	33.19	41.87
Average	33.915	58.152	62.305	70.061	98.637	99.52	98.632	109.592	114.932
The ratio of maximum and minimum demand	3.03	3.65	3.36	4.15	5.16	3.04	3.78	6.97	7.14
Demand for ASCE 41-13 NSP	87.29	107.74	157.64	225.15	303.96	358.7	359.21	493.88	548.10
Roof displacement demands normalized by building height (Avg. Time His.) %	0.226	0.388	0.415	0.234	0.329	0.332	0.219	0.244	0.255
Roof displacement demands normalized by building height (pushover) %	0.582	0.718	1.051	0.750	1.013	1.196	0.798	1.098	1.218

**Figure 21:**
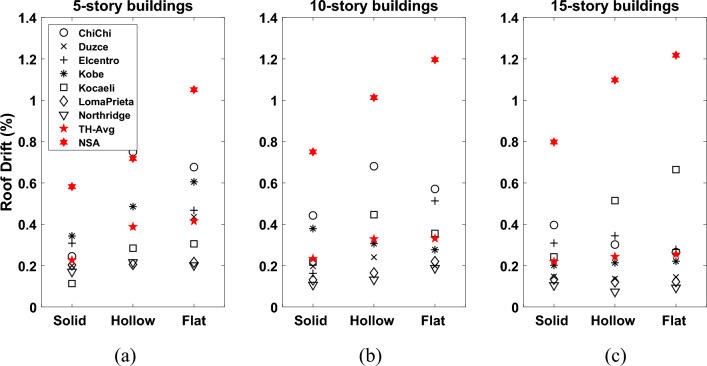
Comparisons of roof drift demands for the (**a**) 5-storey (**b**)10-storey and (**c**)15-storey RC building models with different slab types.

In order to better understand the influence of building heights and slab types on the roof drift ratio demand as well as the behaviour of structures, a comparison of the selected ground motions is presented in Fig. 21. Additionally, the average drift demands for the considered set of records employing NDTHA and inelastic spectral displacement estimated using POA are also plotted in the same figure. The difference between the two demands is more prominent for 10-storey and 15-storey buildings, particularly hollow block and flat slab reinforced concrete buildings*.* The differences between the roof drift ratios calculated by NDTHA and POA are relatively small for the solid slab building models compared to the rest of the models*.* It may also be noted from Fig. 19 that the roof drift ratios have a tendency to decrease for solid slab building models. This is correlated to increase in stiffness capacity of the solid slab buildings.

#### Response modification factor

The response modification factor (R-factor) is one of the most important seismic design factors used in seismic design to reflect the nonlinear behavior of structures. During a major earthquake, the R-factor describes the level of inelasticity in structural systems^59^. Actually, the response modification factor denotes a building's ability to dissipate energy by exhibiting inelastic behavior. Consequently, the structure is designed for much fewer elastic levels of base shear forces than would be required if the structure is remained elastic. Such significant decreases are mainly expected for two reasons: the ductility reduction factor (${R}_{\upmu }$) and the over-strength factor (R_S_). Thus, the response modification factor is defined as:

4 $$R={R}_{\upmu }\times {R}_{S}$$

**Figure 22:**
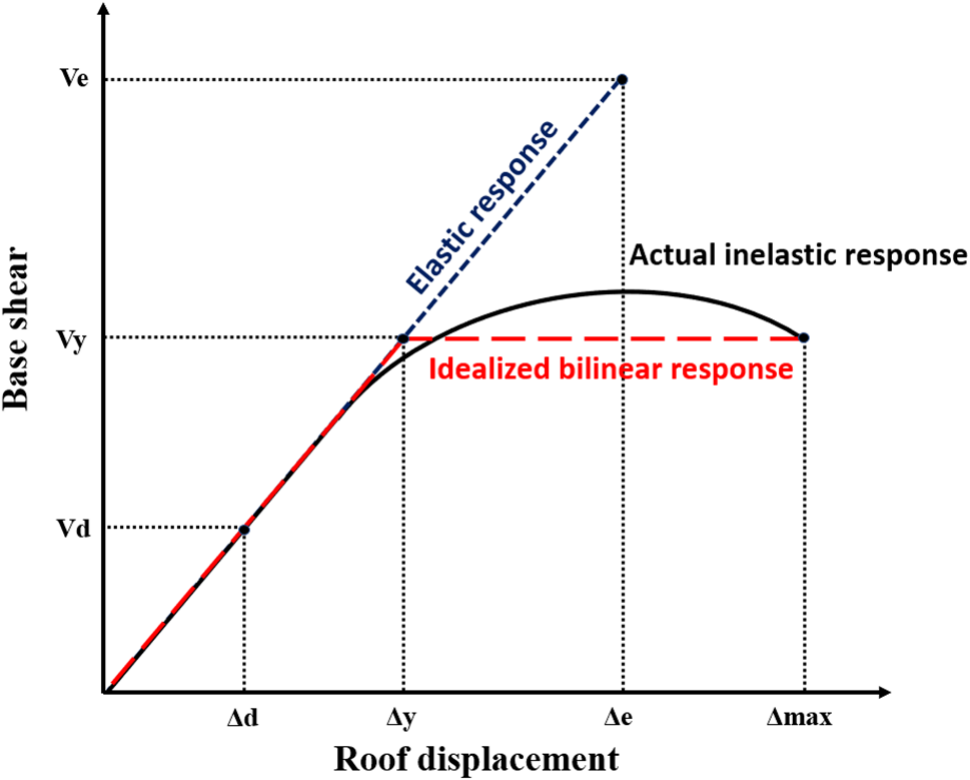
Capacity curve for a structure associated with its bilinear idealization.

${R}_{\upmu }$ is a measure of the strength reduction due to the hysteretic energy. On the other hand, ${R}_{S}$ represents the over-strength introduced by design codes to account for the existence of significant reserve strength that was not considered in the process of design. The R-factor of a building is assessed by conducting a nonlinear static analysis. Figure 22 represents the relation between the base shear against the roof displacement of a structure, which can be calculated by a static pushover loading following the code distribution pattern. In the figure, the real nonlinear behavior of a building is idealized and converted to a bilinear elastic-perfectly plastic relationship (see Fig. 22, the solid line for the real capacity curve and the dotted line for the elastic-perfectly plastic idealization). The initial stiffness of the idealized curve and the position of the zero-stiffness point are calculated by considering that the areas under the actual force–deformation and the idealized curves are equal as proposed in FEMA-356. The ductility reduction factor ${R}_{\upmu }$ is defined as:

5 $${R}_{\upmu }=\frac{{V}_{e}}{{V}_{y}}$$

where ${V}_{e}$ is the base shear considering an elastic response that must be resisted by the structure once it does not show any inelastic behavior; ${V}_{y}$ is the base shear corresponding to the actual yielding of the structure in the idealized behavior. Since ${R}_{\upmu }$ is a function of structural characteristics such as structural ductility ratio µ, fundamental period of vibration (T), and the characteristics of earthquake ground motion^60^, several approximate approaches have been presented for estimating ${R}_{\upmu }$^61^. The parameter $\mu$, which refers to the structural ductility ratio, can be found from the linearized pushover curve for the deformation limits at the selected failure level. The ductility capacity can be calculated as:

6 $$\mu =\frac{{\Delta }_{max}}{{\Delta }_{y}}$$

where ${\Delta }_{\mathrm{max}}$ is the maximum lateral deformation for the first life safety performance in structure and ${\Delta }_{\mathrm{y}}$ is the yield displacement observed there. The analysis of structural performance for numerous buildings subjected to earthquakes have guided to the conclusion that building structures must retain large overstrength to survive without damage under earthquake forces noticeably larger than original design forces.

Overstrength helps structures to withstand sever earthquakes and to reduce the elastic strength demand, as well. The overstrength factor (${R}_{S}$) is calculated as following equation:

7 $${R}_{\mathrm{s}}=\frac{{V}_{y}}{{V}_{d}}$$

where ${V}_{d}$ is the code-prescribed unfactored design base shear. The different base shear levels utilized to describe the two components (${R}_{\upmu }$ and ${R}_{\mathrm{s}}$) for structures of 5, 10 and 15 stories with different types of solid, hollow block and flat slabs are illustrated in Table 5.

**Table 5:** Calculation for components of factor R.

No. story		${V}_{d}$ (kN)	${V}_{y}$ (kN)	${\Delta }_{y}$ (cm)	${\Delta }_{max}$ (cm)	$\mu$	${R}_{\mathrm{s}}$	$R$
5	Solid slab	4254	6025	14.61	87.30	5.97	1.42	8.46
	Hollow block	4621	4673	18.04	107.74	5.97	1.01	6.04
	Flat slab	4442	7505	66.75	157.64	2.36	1.69	3.99
10	Solid slab	5522	13,024	110.69	225.15	2.03	2.36	4.80
	Hollow block	6323	10,246	133.17	303.96	2.28	1.62	3.70
	Flat slab	5755	7418	157.39	358.70	2.28	1.29	2.94
15	Solid slab	6231	10,064	129.52	359.21	2.77	1.62	4.48
	Hollow block	7142	10,297	223.14	493.88	2.21	1.44	3.19
	Flat slab	6516	8735	244.84	548.10	2.24	1.34	3.00

**Figure 23:**
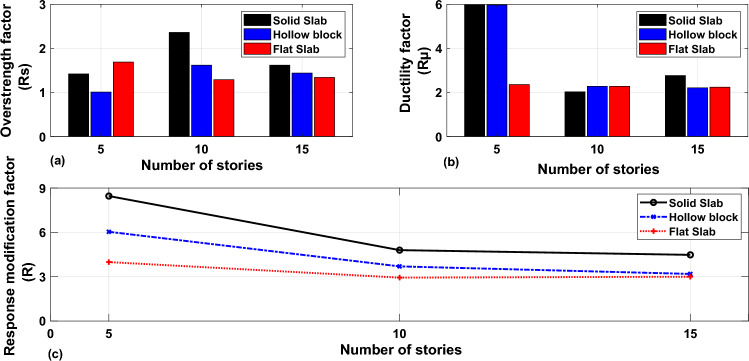
Overstrength, ductility and response modification factors of considered structures with various number of stories.

Response modification factors, ductility and overstrength of the structures with various numbers of stories that were calculated using the methodology presented in “Building models” section are plotted in Fig. 23. The presented results indicate that the overstrength factors are almost constant and equal 1.5 for all building models constructed with various slab types except the 10-storey building model constructed with a solid slab system. The results presented in Fig. 23b show that the structural ductility factors decrease up to a number of 10 stories and remain nearly constant for higher structures. The results of Fig. 23c point out that the R-factors show a gradual decrease as the number of stories increases and remain almost constant in structures with more than 10 stories. This behavior is attributed to the increment in global structural flexibility with the building’s height. This leads to increase in yield-displacement for the structures and consequently reduces the structural ductility factors. Referring to Fig. 23c, it is evident that the R-factors for solid slab building models were higher than those of other models. It was found that the slab type and buildings height have a superior influence on the response modification factors especially for 5-storey building models.

## Conclusions

A detailed study to examine the seismic behavior of nine RC building models with different slab systems and varying heights, namely solid, flat, and hollow block slabs has been conducted. The selected building models are designed and analysed following the requirements of ACI-14, and ASCE7-10 codes respectively. The models were subjected to seven ground motion records from different stations to perform NTHA. While the POA has been performed using the coefficient method following FEMA 440 regulations. The study compares the seismic response demands of NTHA with those of POA for the considered low- mid- and high-rise RC buildings. Additionally, ductility, overstrength and response reduction factors, representing a structure's ability to dissipate energy and reflect the level of inelasticity are evaluated for the RC buildings of different floor systems and storey numbers. Based on the obtained results, the following conclusions are drawn.

- The captured natural periods of the studied floor systems have been found to be substantially different for buildings of the same storey numbers. However, as building heights increase, the influence of floor systems on the induced natural periods becomes more pronounced and noticeable. More specifically, the flat slab system provides the highest ${T}_{n}$ values while the solid slab one provides the lowest leading to the fact that the simultaneous effect of floor systems and heights substantially minimizes or maximizes the induced fundamental periods of structures.
- The covering floor system tends to significantly change the induced seismic base shear coefficient, regardless of the heights of the buildings. However, the increase in storey numbers clarifies the influence of floor systems on the induced base shear coefficients.
- Variations in slab systems provide different simulation results for the displacement and drift profiles. More specifically, floor systems without drop beams, in terms of hollow blocks and flat slabs, produce the highest displacement and drift values due to the significant decrease in the global stiffness of these building models.
- The two methods of analysis employed, in terms of NDTHA and POA, provide almost similar trends regardless of the induced seismic response values.
- Following the POA results of displacement and drift values, hollow blocks and flat slabs suffer extreme loss of strength and failure.
- The seismic code design factors in terms of over-strength, ductility, and response modification show significant changes in values for buildings of low heights and different floor systems. However, as the height increases, the captured values remain nearly unchanged with the change of floor systems with relatively higher values of factors associated with the solid slab system.

The study was conducted exclusively on regular RC structures. The findings of this study can serve as a foundational framework for the design and construction of RC buildings that exhibit various forms of irregularity. Furthermore, the investigation was exclusively conducted for three types of floor systems: solid, hollow blocks, and flat slabs. The present study can serve as a valuable reference for multi-story RC buildings employing base isolation techniques.

## Data Availability

The datasets used and/or analyzed during the current study are available from the corresponding author on reasonable request.
